# Advancing Organ‐on‐Chip Models With a Sacrificial Granular Hydrogel Strategy for Enhanced Permeability and Biomimicry

**DOI:** 10.1002/smtd.202500652

**Published:** 2025-11-05

**Authors:** Hugo R Caires, Óscar Castillo‐Fernández, Núria Sima, Mariana V Magalhães, Andreu Benavent‐Claró, Nil Masó‐Castro, Wanlapa Roobsoong, Carmen Fernandez‐Becerra, Aurora Hernández‐Machado, Hernando A. del Portillo, Cristina C. Barrias

**Affiliations:** ^1^ i3S – Instituto de Investigação e Inovação em Saúde Universidade do Porto Porto 4200‐135 Portugal; ^2^ Malaria and Neglected Parasitic Diseases Program Institute for Global Health (ISGlobal) Barcelona 08036 Spain; ^3^ Institute of Nanoscience and Nanotechnology (IN2UB) University of Barcelona Barcelona 08028 Spain; ^4^ Malalties Infeccioses Institut d'Investigació en Ciències de la Salut Germans Trias i Pujol Badalona 08916 Spain; ^5^ FEUP – Faculdade de Engenharia da Universidade do Porto Porto 4200‐465 Portugal; ^6^ Department of Condensed Matter Physics University of Barcelona Barcelona Spain; ^7^ Mahidol Vivax Research Unit Faculty of Tropical Medicine Mahidol University Bangkok 10400 Thailand; ^8^ Institució Catalana de Recerca i Estudis Avançats (ICREA) Barcelona 08010 Spain; ^9^ INEB – Instituto de Engenharia Biomédica Universidade do Porto Porto 4200‐135 Portugal; ^10^ ICBAS‐Instituto de Ciências Biomédicas Abel Salazar Universidade do Porto Porto 4050‐313 Portugal

**Keywords:** 3D model, infection model, microporogen‐structured hydrogel, porous hydrogel, sacrificial microparticles

## Abstract

Infectious diseases such as malaria, leishmaniasis, and human immunodeficiency virus (HIV) involve pathogens with complex life cycles that span multiple organs, including the bone marrow (BM), a niche for latent or cryptic infections. Studying these hidden stages in patients presents significant technical and ethical challenges, underscoring the need for advanced in vitro models such as organ‐on‐chip (OoC) platforms. While cell‐laden hydrogels can replicate tissue‐like 3D‐microenvironments, their small mesh size may restrict pathogen migration and cell–pathogen interactions, both critical for establishing infection on‐chip. To overcome this limitation, this work develops a “reversed” granular hydrogel strategy that creates interconnected microporosity in hydrogels incorporated into organ‐on‐chip compartments. Sacrificial alginate (ALG) µgels are embedded as porogens in a fibrin–collagen (FIB‐COL) precursor inside a custom BM‐on‐chip and, after crosslinking, are selectively removed by in situ enzymatic/chemical leaching to yield highly porous hydrogels (pFIB‐COL). The pFIB‐COL supports 3D‐cultures of mesenchymal stromal cells, endothelial cells, and erythroblasts. Physical and cellular analyses show reduced flow resistance, enhanced particle and cell permeation, more uniform cell distribution and improved endothelial network formation compared with native FIB‐COL. This versatile strategy is readily adaptable to other hydrogel systems, providing a valuable tool for the faithful modeling of infection processes in biomimetic 3D‐microenvironments within OoC devices.

## Graphical abstract

A reversed granular hydrogel strategy creates highly porous 3D matrices within bone marrow (BM)‐on‐chip devices. Sacrificial alginate microgels generate interconnected microporosity that enhances nano/microparticle and cell mobilitymimicking pathogen agents—from the perfused vascular channel into the BM compartment, while preventing cell sedimentation and promoting 3D morphogenesis and primitive microvasculature formation. This adaptable strategy offers a valuable tool for faithful infection modelling on‐chip.

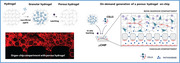

## 1 Introduction

Several hematologic diseases directly affect the bone marrow, the primary site of erythropoiesis, by disrupting the production of red blood cells, white blood cells, and platelets. Among these, infectious diseases such as malaria, leishmaniosis, human immunodeficiency virus (HIV), and tuberculosis – responsible for millions of clinical cases each year – can establish latent, sub‐patent chronic infections in this tissue, thus evading immune responses and drug treatments.^[^ ^1^, ^2^, ^3^, ^4^ ^]^ A comprehensive understanding of these disease stages will support the development of novel strategies for their detection, control, and eradication, ultimately advancing curative therapies. However, investigating these hidden reservoirs in patients remains ethically and technically challenging, underscoring the urgent need for advanced in vitro models that replicate human organ environments.

Organ‐on‐chip (OoC) technology has emerged as a powerful tool for creating physiologically relevant in vitro models.^[^ ^5^, ^6^, ^7^, ^8^, ^9^ ^]^ These microfluidic devices mimic key features of human organs, such as compartmentalization, tissue–tissue interfaces, and dynamic perfusion; enabling controlled studies of complex biological processes, including inter‐organ cross‐talk.^[^ ^5^, ^6^, ^7^, ^8^, ^9^ ^]^ Beyond emulating organ‐level anatomy/physiology, OoC platforms are transforming disease modeling. They enable the recreation of diverse disease and genetic disorder settings, as well as host–microbiome/pathogen and immune interactions, providing a means to investigate human clinical responses to drugs, infectious agents, and other insults.^[^ ^5^, ^6^, ^7^, ^8^, ^9^ ^]^ By capturing patient‐specific biology with unprecedented precision, OoC systems are further driving advances in precision medicine, biomarker discovery, and therapeutic development.^[^ ^5^, ^6^, ^7^, ^8^, ^9^ ^]^ A key regulatory milestone was recently set by the U.S. Food and Drug Administration (FDA), which outlined a roadmap to phase out animal testing for monoclonal antibodies and other biologics, endorsing OoC and related New Approach Methodologies (NAMs).^[^ ^10^ ^]^

In bone marrow (BM)‐on‐chip (BM‐on‐chip) systems, the BM is typically represented by a cell‐laden hydrogel compartment connected to a vascular channel that simulates microcirculation.^[^ ^5^, ^6^ ^]^ Replicating the heterogeneous BM microenvironment for infection research, however, presents unique challenges.^[^ ^8^ ^]^ Beyond incorporating multiple cell types, such as hematopoietic stem cells, mesenchymal stromal cells (MSCs), and endothelial cells into a 3D matrix, it is also necessary to introduce infectious agents and recapitulate the infection process. In this context, the vascular channel must mimic the passage of pathogens in the bloodstream, enabling their migration into the BM compartment. Yet, while cell‐laden hydrogels can emulate tissue‐like 3D microenvironments, their small mesh size (typically 10–1000 nm^[^ ^11^, ^12^ ^]^) can hinder the migration of pathogens, such as viruses (20–200 nm) and free parasites (2–3 µm), as well as infected cells (7–20 µm).This mechanical confinement may therefore restrict interactions between infectious agents and hydrogel‐embedded cells, imposing significant constraints in modeling infection dynamics in BM‐on‐chip platforms.

Increasing hydrogel porosity has long been a key goal in tissue engineering, often achieved through established techniques such as freeze‐drying, salt/porogen templating, and gas foaming,^[^ ^13^, ^14^, ^15^ ^]^ among others. However, these methods are generally unsuitable for in situ (on‐chip) hydrogel crosslinking and are often not cytocompatible, limiting their application in closed‐chamber organ‐on‐chip (OoC) devices containing living cells. Some hydrogel systems rely on chemical and/or enzymatic degradation to create more permissive 3D matrices that facilitate cellular infiltration and activity.^[^ ^16^, ^17^, ^18^ ^]^ Yet, the degradation process is often unpredictable and may require extended culture periods to generate sufficiently large pores, making it impractical for short‐term studies of host‐pathogen interactions, and incompatible with the dynamic, on‐demand creation of predefined porosity.

A promising alternative strategy is the use of granular hydrogels, which are composed of densely packed microgel particles (granules) that assemble into a 3D scaffold. Unlike conventional bulk hydrogels, where porosity is defined by the polymer network, granular hydrogels inherently contain larger, interconnected pores formed by the interstitial spaces between individual particles.^[^ ^19^, ^20^, ^21^, ^22^, ^23^, ^24^ ^]^ This architecture facilitates fluid transport, promotes cell migration, and increases available surface area for cell attachment. Consequently, granular hydrogels have been widely explored in regenerative medicine as injectable porous biomaterials that support cell colonization, vascularization, and tissue regeneration.^[^ ^19^, ^20^, ^21^, ^22^, ^23^, ^24^ ^]^

Here, we introduce a “reversed” granular hydrogel strategy, where sacrificial microgels (µgels) are combined with the primary hydrogel component to act as microporogens, rather than serving as scaffolding material. We selected alginate‐based (ALG) µgels due to their bioinert nature, which prevents unwanted cellular activation upon µgel disassembly, unlike protein‐based materials^[^ ^25^ ^]^ that may elicit cellular responses. As a proof of concept, the ALG µgels were incorporated into a fibrin‐collagen (FIB‐COL) precursor solution, a widely used matrix for 3D cell culture. The resultant granular hydrogel (gFIB‐COL) was crosslinked, entrapping the µgels within the FIB‐COL network. A subsequent mild enzymatic/chemical leaching step selectively removed the µgels, generating an interconnected porous network within the primary hydrogel matrix (pFIB‐COL). This approach allows precise control over pore size and density, while maintaining cell viability and the structural integrity of the surrounding FIB‐COL network. To validate the approach, we designed a BM‐on‐chip system featuring a perfusable vascular channel to simulate infected blood flow and a BM compartment filled with the in situ crosslinkable gFIB‐COL. We show that by introducing well‐defined porosity through µgel leaching, our approach significantly improved hydrogel permeability, enhancing fluid flow and facilitating the transport of model particles and cells from the vascular channel into the BM compartment. This strategy, broadly applicable to other hydrogel systems, provides a valuable tool for applications requiring efficient particle and cell transport through 3D matrices, such as infection modeling.

## 2 Results and Discussion

### 2.1 Alginate Microgels Present Tunable Size and Uniform Round‐Shape

**Figure 1:**
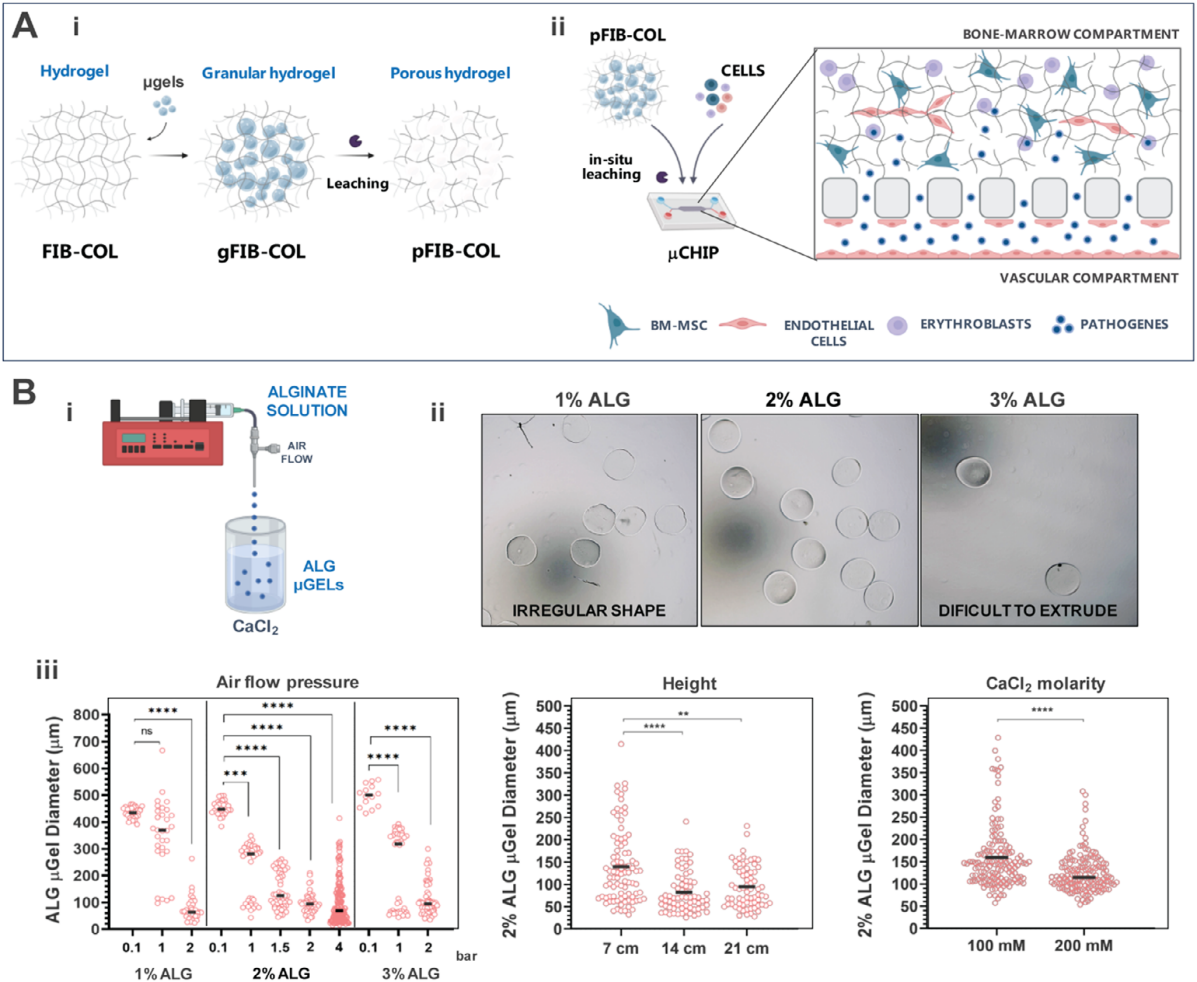
A ‘reversed’ granular hydrogel strategy using sacrificial ALG µgels to enhance the porosity of FIB‐COL hydrogels. A) Graphical representation of micropore formation within bulk FIB‐COL hydrogel using sacrificial ALG µgels. i. Sacrificial ALG µgels are embedded in the FIB‐COL precursor solution prior to crosslinking forming gFIB‐COL hydrogel. The on‐demand enzymatic/chemical leaching of ALG µGels creates an open microporosity within the densely packed FIB‐COL hydrogel (pFIB‐COL). ii. The gFIB‐COL can be combined with cells, loaded into a microfluidic device and leached on‐chip. Created with BioRender.com. B) Fabrication of ALG µgels by (i) Co‐axial Air Flow method coupled with external gelation in a CaCl_2_ crosslinking bath. (ii) The ALG solution concentration impacts the yield and spherical shape of the µgels under the same extrusion settings. (iii) Adjusting the extrusion parameters, including the air flow pressure, height from the needle to crosslinking bath, and molarity of CaCl_2_ crosslinking bath allows controlled production of ALG µgels with diameter in the range 50–100 µm. All results are expressed as the median (n = 14–160 µgels, from 3 independent experiments). Statistical analysis of all quantitative data was performed either via Kruskal‐Wallis test with post hoc Dunn's comparison tests or via Mann‐Whitney test. ** (*p* < 0.01), *** (*p* < 0.001), and **** (*p* < 0.0001).

The “reversed” granular hydrogel approach, illustrated in **Figure** **1**A‐i, uses sacrificial microgels (µgels) as microporogens. This method introduces well‐defined porosity through µgel leaching, greatly improving bulk hydrogel permeability. The granular hydrogel can be loaded into a chip and micropores can be generated in situ to facilitate fluid/particle transport from the vascular channel into the bone marrow (BM) compartment (Figure 1A‐ii). Sacrificial ALG µgels were produced via high‐throughput droplet extrusion into a CaCl_2_ crosslinking bath under co‐axial air flow^[^ ^26^, ^27^ ^]^ (Figure 1B‐i). Hydrogels form through ionic crosslinking when ALG molecules, composed of negatively‐charged mannuronic (M) and guluronic (G) acid units, interact with divalent cations like calcium (Ca^2^⁺). The calcium ions bind selectively to the G‐blocks of the ALG chains, creating “egg‐box” junctions that link polymer chains together.^[^ ^28^ ^]^ This reversible crosslinking mechanism results in the formation of a stable hydrogel 3D network. To trigger external gelation, ALG solutions are extruded as droplets into a Ca^2^⁺‐containing crosslinking bath. Upon contact with the bath, gelation occurs spontaneously at the droplet surface, rapidly forming a µbead‐like structure while preserving the spherical shape of the droplets. Subsequently, the crosslinking process extends inward, gradually solidifying the entire µbead to achieve stable and uniform µgels. Parameters such as ion concentration, ALG concentration, and extrusion conditions can be adjusted to precisely control the µgel size and morphology, enabling customization. Here, the target was to achieve µgels in the range of 50–100 µm, which should allow unrestricted permeation by nano/micro particles and cells, while also facilitating cellular interactions and the formation of primitive vascular networks. To this end, we used a small needle size (0.09 mm) and systematically investigated the effects of ALG concentration (1–3 wt.%), air flow pressure (0.1–4 bar), needle height above the crosslinking bath (7, 14, and 21 cm), and molarity of the CaCl_2_ solution (100 or 200 mM). As shown in Figure 1B‐ii, the ALG concentration did not significantly impact the size of the gels. However, a 1 wt.% concentration resulted in more irregularly shaped µgels, while increasing the concentration to 3 wt.% made extrusion challenging, reducing the yield of µgel production. The best results, balancing shape regularity and ease of extrusion, were achieved with a 2 wt.% ALG solution. The air flow pressure emerged as the most impactful parameter, with µgel diameter significantly decreasing as pressure increased (Figure 1B‐iii). For the 2 wt.% ALG solution, the diameter was ≈500 µm at 0.1 bar and reduced to ≈100 µm at 2 bar. Further increasing the pressure to 4 bar showed minimal additional effect. Increasing the needle‐to‐bath distance also slightly decreased µgel size, and while no significant differences were found for distances of 14 and 21 cm, we decided to setup the system at 21 cm. Finally, increasing the CaCl_2_ molarity from 100 to 200 mM also slightly reduced the µgel size. Overall, by combining all the optimized parameters (2 wt.% ALG, 2 bar, 21 cm, 200 mM CaCl_2_), we successfully produced µgels within the target size range and with a uniform spherical shape and a very smooth surface. This is essential for generating (interconnected)micropores with predictable dimensions in hydrogel matrices, without damaging their native‐like fibrillar structure. These µgels can be easily fabricated using commercially available ALG and very simple equipment, which facilitates the widespread use of our approach. Notably, the size/shape of the µgels remained unchanged when using ALG modified with functional groups for fluorescent labeling (data not shown), which enable effective visualization/tracking µgels fate within the hydrogel network before and after leaching, for process optimization.

### 2.2 Bone Marrow‐on‐a‐Chip Device

The microfluidics platform used in this study was designed with two main objectives: 1) to accommodate a substantial hydrogel volume for the cell compartment, which facilitates down‐streaming analysis, and 2) to enable injection of the FIB‐COL pre‐gel mixture into the BM compartment while avoiding leakage into the vascular compartment (Figure SI‐1, Supporting Information). To address these challenges, we used a 3D printer to overcome the limitations associated with cross‐sectional constraints and the high costs of standard photolithography techniques.^[^ ^6^ ^]^ The custom‐made device features a BM compartment with a cross‐section of 1×1 mm, capable of accommodating up to 20 µL of hydrogel, alongside a lateral vascular channel measuring 0.2 × 1 mm in cross‐section. Both channels are interconnected by a series of 15 slits, spaced 0.6 mm apart, each with a cross‐section of 0.1 mm × 1 mm and length of 0.6 mm. These slits enable exchange of materials between the two compartments while providing sufficient fluidic resistance to allow manual injection of the pre‐gel mixture without spilling into or blocking the vascular channel. The intercompartmental exchange includes diffusion of soluble molecules as well as migration of biological entities of various sizes (e.g., viruses, parasites, and infected cells). In our study, these transport capabilities were validated using model particles and representative cell types.

GreyPro resin was chosen for mold fabrication because it allowed reliable PDMS polymerization and easy demolding without deformation or breakage. Using this resin, the FormLabs printer successfully reproduced the required fine cross‐sections, however, as expected with the laser‐based technology, rounded edges were observed at the channel–slit junctions (Figure S1, Supporting Information) resulting in an average slit width of ≈0.15 mm. Despite this limitation, the final PDMS slits geometry (Figure S1, iv, Supporting Information) remained sufficiently narrow to prevent leakage of the FIB‐COL pre‐gel mixture during injection and avoided obstruction of the lateral vascular channel.

### 2.3 ALG µgel Content Dictates the Microporous Structure of pFIB‐COL Hydrogel

**Figure 2:**
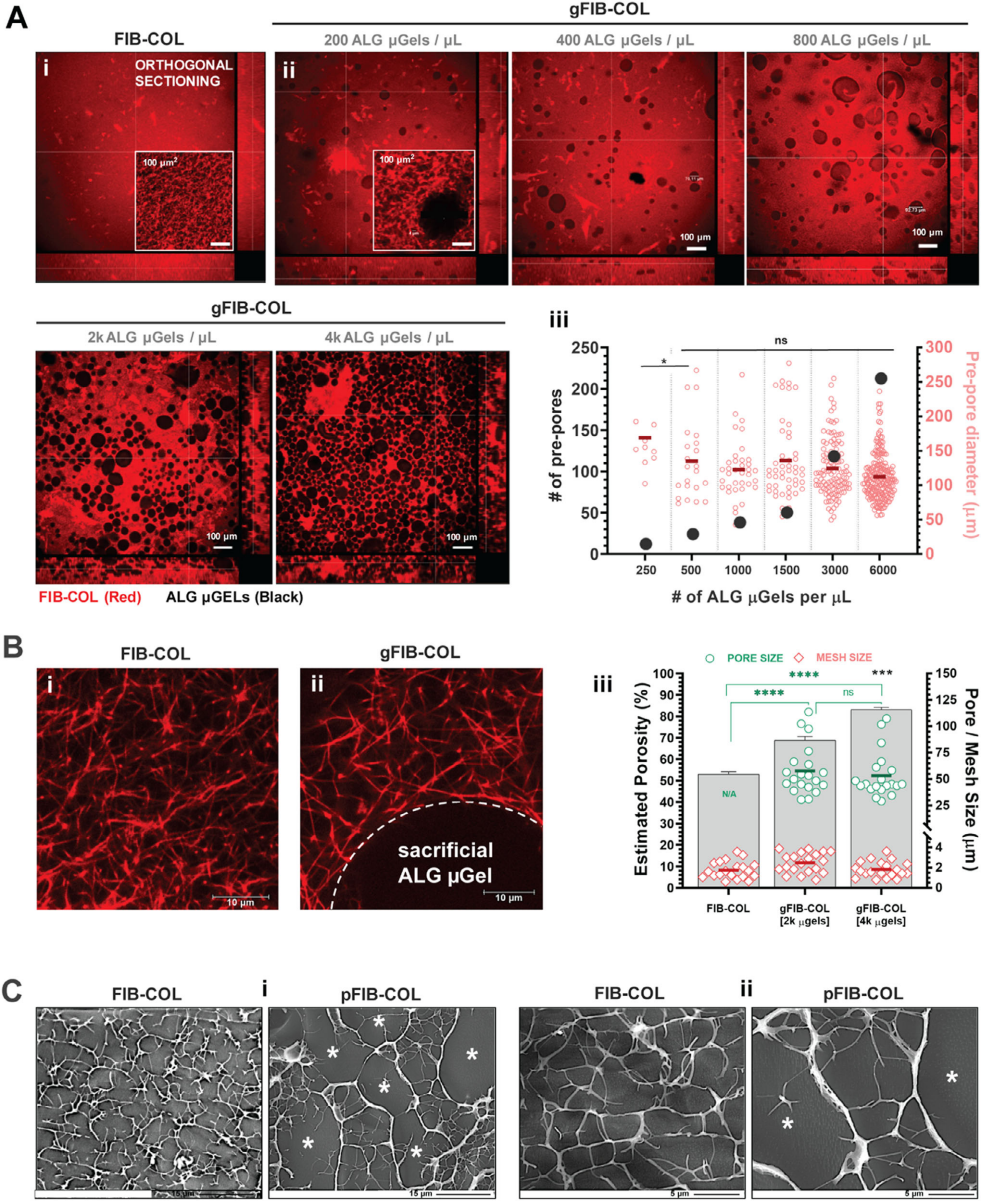
The microporosity of gFIB‐COL hydrogels can be tuned by varying the amount of ALG µgels. A) Orthogonal sectioning of confocal microscopy images showing the (i) FIB‐COL hydrogel which present a dense fibrillar mesh (red regions, NHS‐rhodamine), and the (ii) effect of incorporating increasing amounts of ALG µgels (unstained black regions). Interconnected porosity is observed from 2000 ALG µgels per µL onward in the orthogonal projections. (iii) Image‐based quantification of the mean number and diameter of pores within gFIB‐COL as a function of the amount of ALG µGels per imaged section. B) Higher magnification confocal images illustrating the fibrillar mesh size of (i) FIB‐COL hydrogels and (ii) gFIB‐COL pre‐mixed with ALG µgels (white dotted line). No alteration in native FIB‐COL fibrillar mesh size was observed upon incorporation of ALG µgels integration. (iii) Image‐based quantification of estimated porosity (left axis, grey bars, black *** gFIB‐COL [4k µgels] versus FIB‐COL porosity) and FIB‐COL fibrillar mesh size (right axis, red dots, not significant for all cases) or large micropores for different amounts of ALG µgels (right axis, green dots, green brackets **** or not significant). All results are expressed as the mean ± SEM (n = 5–20 images analyzed from at least 3 independent hydrogels). C) CryoSEM images of FIB‐COL and pFIB‐COL hydrogels at different magnifications: i – Scale bar 15 µm, and ii – Scale bar 5 µm (* represents larger pores formed upon leaching of ALG µgels. Statistical analysis of quantitative data was performed either 2‐way ANOVA with post hoc Turkey's comparison test (A‐iii) and Kruskal‐Wallis test with post hoc Dunn's comparison tests (B‐iii). * (*p* < 0.05), *** (*p* < 0.001), and **** (*p* < 0.0001).

Having established the optimal conditions for producing ALG µgels, we next focused on optimizing their integration within FIB‐COL hydrogels. To achieve this, we tested varying concentrations of ALG µgels per volume of FIB‐COL precursor to determine the amount required for generating interconnected porosity throughout the hydrogel bulk (**Figure** **2**). Using amine‐reactive fluorescent labeling, we observed that native FIB‐COL hydrogels consist of fibrin fibrils with sparse spots of dense collagen fibers, with a tight mesh size of ≈2–3 µm (Figure 2A‐i). We tested ALG µgel densities ranging from 200 to 6000 µgels per µL of FIB‐COL precursor and observed that interconnection was only achieved when more than 2000 µgels were used (Figure 2A‐ii). As predicted, image‐based quantification in confocal microscopy image sections revealed a linear relationship between the theoretical ALG µgel density and the total space occupied by µgels (Figure 2A‐iii). Notably, the integration of ALG µgels did not significantly alter the native fibrillar of the FIB‐COL network (Figure 2B‐i,ii), suggesting that the presence of ALG µgels did not interfere with the crosslinking process and preserved the original structure of the primary hydrogel component. The gFIB‐COL hydrogels containing 2000 or 4000 µgels (per µL) presented pre‐pores with a mean diameter of ≈65 µm (Figure 2B‐iii). As expected, the higher number of ALG µgels resulted in the enhancement of the bulk hydrogel's estimated porosity, reaching over 80% (4K µgels). In contrast, the native FIB‐COL hydrogel, with a mesh size of 2–3 µm, had an estimated porosity of 53%. Taken together, these findings demonstrate that the amount of sacrificial ALG µgels integrated within FIB‐COL dictates the pre‐pore structure, with higher densities of ALG µgels being essential for ensuring interconnectivity. Depending on the application, the best compromise between porosity and scaffolding volume for cell growth should be selected. CryoSEM images (Figure 2C i,ii) further illustrate the fibrous nature of the original FIB‐COL network and the significant increase in hydrogel porosity obtained upon leaching of the ALG µgels (please see Section 2.4 for details on the leaching process). Notably, although we did not characterize their bulk mechanical properties, the preservation of the native fibrillar network in pFIB‐COL hydrogels compared with FIB‐COL strongly suggests that the local mechanical cues most relevant to the behavior of embedded cells remain largely unchanged, which is the desired outcome.

### 2.4 Leaching of Sacrificial ALG µgels Generates Highly Porous pFIB‐COL Hydrogels

**Figure 3:**
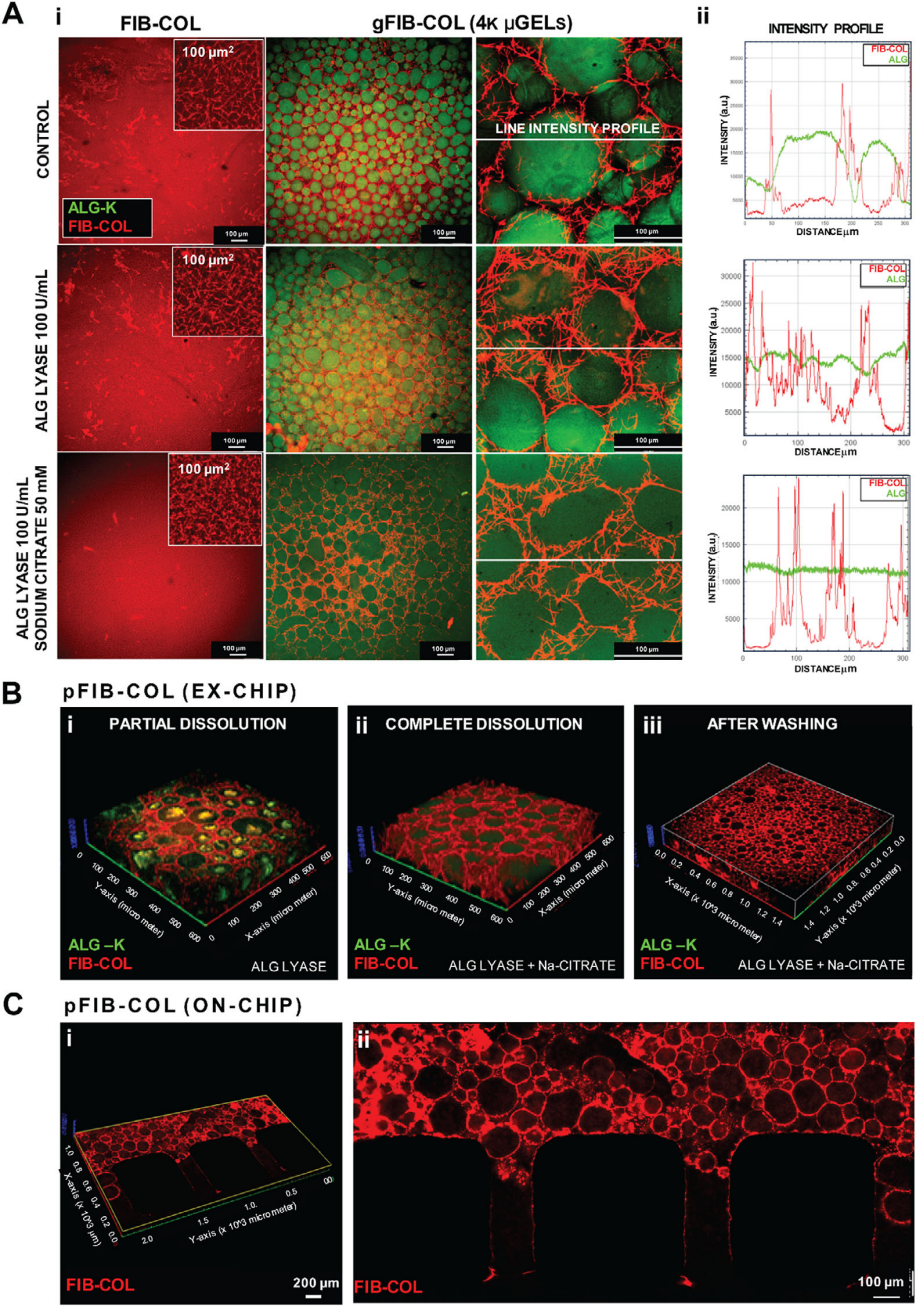
Leaching of sacrificial ALG µgels generates highly porous pFIB‐COL hydrogels. A) Effect of leaching in the (i) the FIB‐COL and granular gFIB‐COL hydrogels (with 4k ALG µgels), treated with NaCl 0.9% (control), 100 U mL^−1^ ALG lyase (enzymatic), or ALG lyase + 50 mM sodium citrate (enzymatic/chemical). The FIB‐COL fibrillar structure is labeled in red and the ALG is labeled in green. (ii) Image‐based quantification (mean line intensity across dotted white line) illustrating the impact of the different treatment conditions on the microstructure of gFIB‐COL hydrogels, showing ALG µgels disintegration (plateau green line) or fibrillar FIB‐COL mesh (spiked red line). B) Ex‐chip leaching in gFIB‐COL hydrogels: 3D reconstruction of the porous structure (FIB‐COL mesh in red, ALG in green) after 30 min of (i) enzymatic, (ii) enzymatic/chemical treatment, and (iii)enzymatic/chemical treatment after washing. The uniform distribution of the micropores is highlighted in 3D sectioning. C) On‐chip leaching in gFIB‐COL hydrogels, after loading into the microfluidic device (BM‐compartment) and in situ crosslinking. (i) Leaching was performed using the combined enzymatic/chemical (30 min), with the solution being perfused though the vascular channel. (ii) 3D reconstruction and confocal image plane showing the open porosity of the pFIB‐COL hydrogel.

We optimized the ALG µgel leaching process to create open and interconnected porosity within gFIB‐COL hydrogels. Different strategies were tested, including: i) selective enzymatic digestion of ALG µgel chains into smaller fragments using ALG lyase; and ii) enzymatic digestion combined with sodium citrate treatment, a chelating agent that disrupts the Ca^2+^‐mediated crosslinking of the µgel network (**Figure** **3**). To track the leaching process and ensure efficient ALG removal, we conducted these experiments using µgels composed of ALG chemically modified with cyclooctyne groups (ALG‐K) and labeled with a fluorescent azide‐tag via strain‐promoted‐azide alkyne cycloaddition (SPAAC).^[^ ^29^ ^]^ Treatment of the gFIB‐COL hydrogel (containing 4K ALG µgels, to maximize porosity) with ALG lyase, with or without sodium citrate, did not alter its native structure. Enzymatic digestion with ALG lyase led to partial disassembly of the µgels, as indicated by the fading ALG‐associated fluorescence signal at the µgel locations compared to the untreated control (Figure 3A‐i,ii). The leaching efficiency improved when ALG lyase treatment was combined with sodium citrate, as seen by the flattened fluorescence signal across the entire field of view. Figure 3B shows that partial or complete disassembly of µgels, depending on the treatment, occurred in a uniform pattern across the x/y/z planes of the bulk gFIB‐COL hydrogel (i.e., in a well, outside the chip), as evidenced by the 3D image reconstructions. Similar results were observed on‐chip (Figure 3C). The gFIB‐COL hydrogel precursor was successfully injected into the BM compartment without leakage into the vascular channel and was efficiently and homogenously crosslinked in situ. Leaching was initiated by infusing a solution containing ALG lyase and sodium citrate into the vascular channel, resulting in a hydrogel with a uniform, interconnected porous structure, hereafter referred to as pFIB‐COL. Notably, the leaching process can be triggered at any point during culture, enabling the creation of porosity “on demand” and providing greater experimental design flexibility. This offers a notable advantage over other granular hydrogel approaches, where thermally sensitive µgels naturally disassemble at 37 °C, limiting control over porosity formation.^[^ ^25^ ^]^

### 2.5 The pFIB‐COL Hydrogels Offer Less Resistance to Fluid Flow

**Figure 4:**
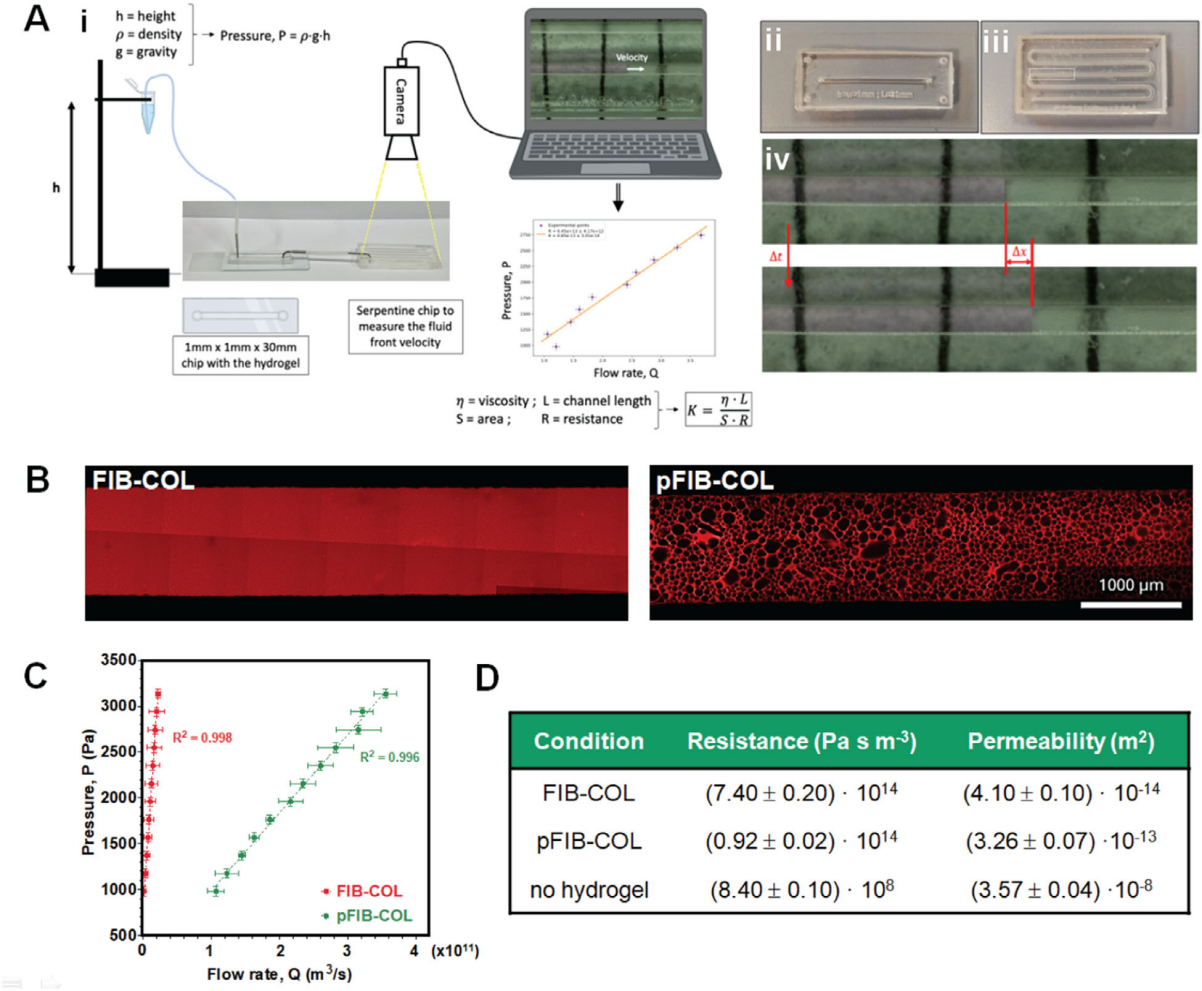
The pFIB‐COL hydrogels offer less resistance to fluid flow compared to FIB‐COL. A) (i) Schematic representation of the system used to assess the impact of increased porosity on FIB‐COL hydrogel permeability. The fluidic system consists of a reservoir containing cell medium at a given height (h). The medium flows through tubing (with length *l_t1_* = 40 cm and radius *r_t_* = 0.25 mm) into a chip loaded with hydrogel. After passing through the hydrogel, the medium flows through another tubing (with length *l_t2_* = 3 cm) before reaching a serpentine chip, where the flow rate is measured using image‐based analysis. (ii) Image of a chip loaded with hydrogel. (iii) Serpentine chip. (iv)Images showing the liquid front movement within the serpentine chip at two different time points. Front velocity is determined using *v = Δx/Δt*. B) FIB‐COL (left) and pFIB‐COL (right) hydrogels stained with NHS‐Rhodamine. C) Quantification of pressure versus flow rate in FIB‐COL and pFIB‐COL hydrogels. D) Summary table of measurements of resistance and permeability in FIB‐COL and pFIB‐COL hydrogels.

Next, we evaluated whether pFIB‐COL hydrogels effectively offered an improvement in permeability on‐chip across multiple scales compared to standard FIB‐COL hydrogels. To address this, we have set up a microfluidic system to assess the resistance of pFIB‐COL hydrogels to fluid flow, in comparison with FIB‐COL hydrogels (details provided in the Materials & Methods section) (**Figure** **4**A). In brief, this system features a reservoir holding cell medium at a specified elevation, from which the medium passes through a silicone tube into a hydrogel‐filled chip. After traversing the hydrogel, the medium exits via a second tube into a serpentine chip, where the flow rate is determined, enabling calculation of system resistance using the fluidic Ohm's law based on the pressure gradient from the reservoir height and the observed flow rate. The microfluidic devices loaded with hydrogel were analyzed by imaging to assess the integrity of the FIB‐COL matrix (Figure 4B). Following crosslinking, the µgels were successfully leached out, generating interconnected voids throughout the pFIB‐COL scaffold. Due to the small scales involved, minimal liquid volumes were required to assess the hydrogel's fluidic properties. Fluidic resistance (*R*) represents the opposition to fluid flow within the system, influenced by the hydrogel's geometrical structure and the viscosity of the fluid. When a pressure gradient is applied across the hydrogel, the resulting flow rate is governed by this resistance. In contrast, permeability (*K*) is an intrinsic property of the hydrogel's porous microstructure, reflecting the ease with which fluids traverse the material. It is independent of fluid properties, system geometry, or medium volume. Notably, a clear difference was observed between flow resistance slopes in native FIB‐COL hydrogel experiments compared to the leached pFIB‐COL hydrogels (Figure 4C). As described in equation 1, the slope corresponds to the material's resistance to media flow:

(1) P=R·Q

where the pressure (P) is proportional to the resistance (R) and the flow rate (Q). The leached pFIB‐COL hydrogel exhibited a significantly lower resistance to fluid flow compared to FIB‐COL hydrogels, with resistance reduced by ≈15‐fold. Since resistance is not an intrinsic property of the porous medium, it is essential to evaluate permeability, which is a material‐specific property and inversely proportional to resistance, as defined by Equation 2:

(2) $$K=\left(\eta \cdot L\right)\;/\;\left(S\cdot R\right)$$

where K represents permeability, η is the dynamic fluid viscosity, L is the length of the hydrogel, S is the cross‐sectional area, and R is the resistance.

As shown in Figure 4D, the permeability of pFIB‐COL hydrogels significantly increased compared to FIB‐COL, as expected. Figure 4D also highlights a notable decrease in the resistance of pFIB‐COL hydrogels in this microfluidic system. Additionally, pFIB‐COL demonstrated higher permeability than FIB‐COL. This improvement arises from the formation of additional void spaces within the hydrogel's internal architecture. These newly formed pores reduce the overall resistance to fluid flow, enabling more efficient liquid movement through the material. These findings further support the idea that increased porosity enhances permeability, making the hydrogel a more effective medium for fluid transport.

### 2.6 Permeation of Model Particles/Cells is Greatly Improved in pFIB‐COL

**Figure 5:**
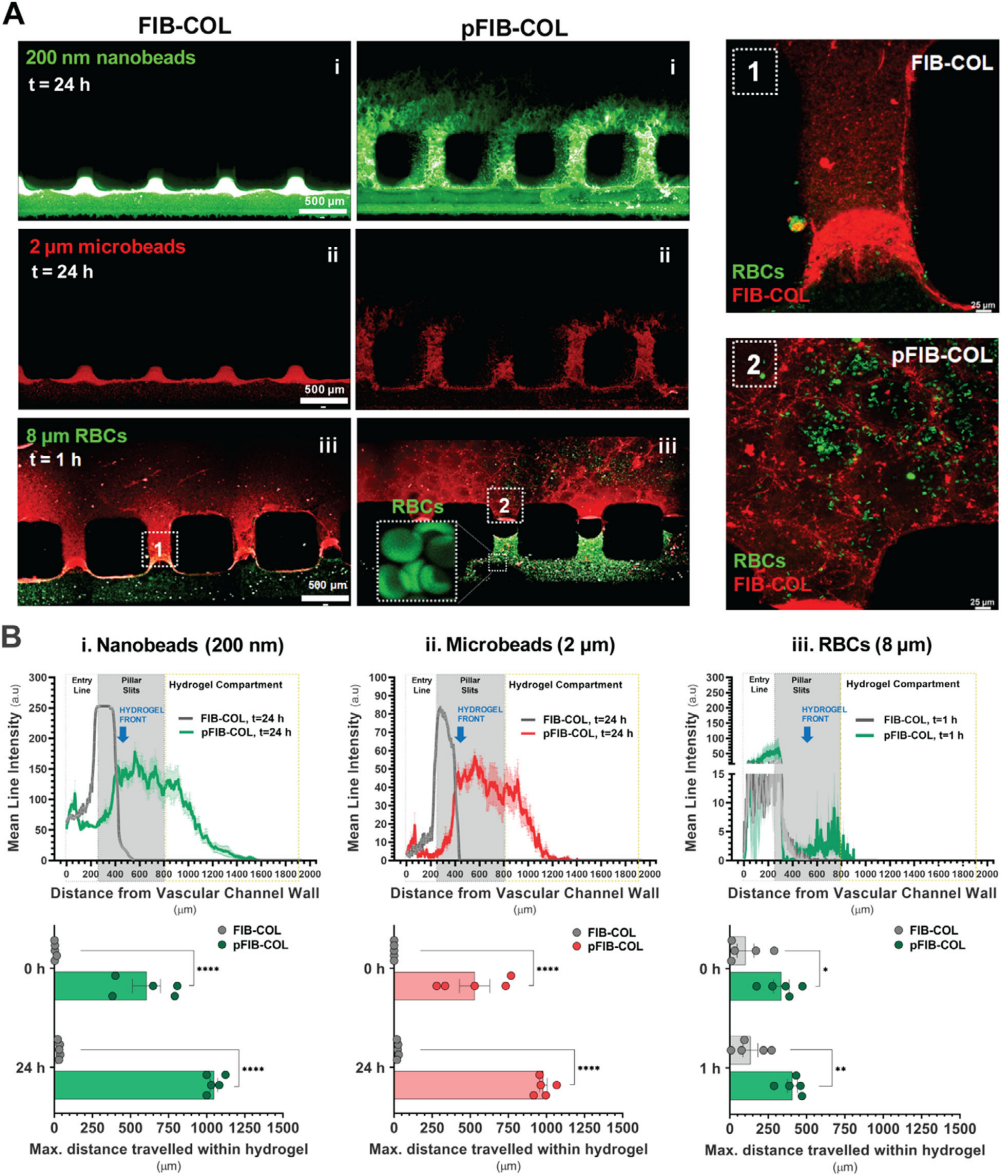
Permeation of model particles of different sizes in pFIB‐COL compared to FIB‐COL. A) The FIB‐COL (left) and pFIB‐COL (right) hydrogel permeability was studied using fluorescent model particles of (i) 200 nm (green) or (ii) 2 µm (red) and (iii) fluorescently labeled human RBCs of 8–10 µm (green). Scale bar, 500 µm. The model particles were infused via the vascular channel for 5 min ON‐OFF cycles (10 µL min^−1^) during 30 min, followed by 24 h static incubation to favor gravity driven crossing of particles trough the hydrogel. The 8–10 µm diameter human RBCs were continuously infused at the same rate during 1 h at 37 °C. Representative^[^ ^1^ ^]^ and^[^ ^2^ ^]^ inserts showing magnified photographs of RBCs permeation across the hydrogel (FIB‐COL fibrillar network in red) in regions next to the pillar slits. B) Image‐based quantification of the mean line intensity (top) and maximum distance travelled by the infused particles: (i) 200 nm, (ii) 2 µm or (iii) fluorescently labeled human RBCs, through the hydrogel (bottom) across multiple pillar slits covering the entry line in the vascular channel (200 µm wide per 2 mm long), the pillar slits and all the hydrogel compartment chamber. Statistical analysis of maximum distance travelled by infused particles per each slit was performed by 2‐way ANOVA with post hoc Turkey's comparison test. * (*p* < 0.05), ** (*p* < 0.01), and **** (*p* < 0.0001).

We then tested the permeability of pFIB‐COL hydrogels to a range of elements with physiological relevance to infection studies. Fluorescently labeled model particles and cells including: i) 200 nm nanobeads (virus‐sized), ii) 2 µm microbeads (parasite‐sized), and iii) human RBCs (≈8 µm), were suspended in culture media and infused into the vascular channel at flow rates of 10 µL min^−1^ for ON‐OFF cycles of 5 min each during 30 min (beads) or continuously for 1 h (RBCs). Chips were imaged immediately upon first passage (T0) via confocal microscopy and after 24 h (beads) or 1 h after infusion (RBCs). As shown in **Figure** **5** i–iii, the engineered pFIB‐COL hydrogel facilitated the permeation of the model particles deep into the BM‐compartment. In contrast, the unmodified FIB‐COL hydrogel acted as a more restrictive physical barrier, causing particles to accumulate at the fluid‐matrix interface between pillars. Additionally, while RBCs traveled no more than 50–100 µm into the dense FIB‐COL hydrogel, they penetrated significantly deeper within the pFIB‐COL matrix, with several observed at the farthest end of the BM‐compartment. Notably, RBCs appeared to preferentially migrate through the void spaces, accumulating within pores in different regions of the hydrogel (Figure 5A, right column inserts 1 and 2). As depicted in Figure 5B quantification of mean line intensity (top) and maximum distances travelled by the infused particles through the hydrogel (bottom), across multiple, randomly distributed pillar slits further illustrated the permeability differences among the hydrogels and across distinct particle sizes. Together, these data demonstrate that highly porous pFIB‐COL hydrogels can be engineered on‐chip, showing significantly enhanced permeability and exchange of materials from the vascular into the BM compartment, compared to densely packed FIB‐COL hydrogels. Significantly, while this study used model particles to assess permeability under controlled conditions, it is anticipated that real biological entities – such as viruses, parasites, or infected RBCs – would exhibit more complex behaviors, including tropism and enhanced motility. This could lead to even more efficient penetration and distribution within the porous matrix, further improving cell‐pathogen dynamic interactions.

### 2.7 The Integration and Leaching of ALG µgels from FIB‐COL do not Compromise Cell Viability

**Figure 6:**
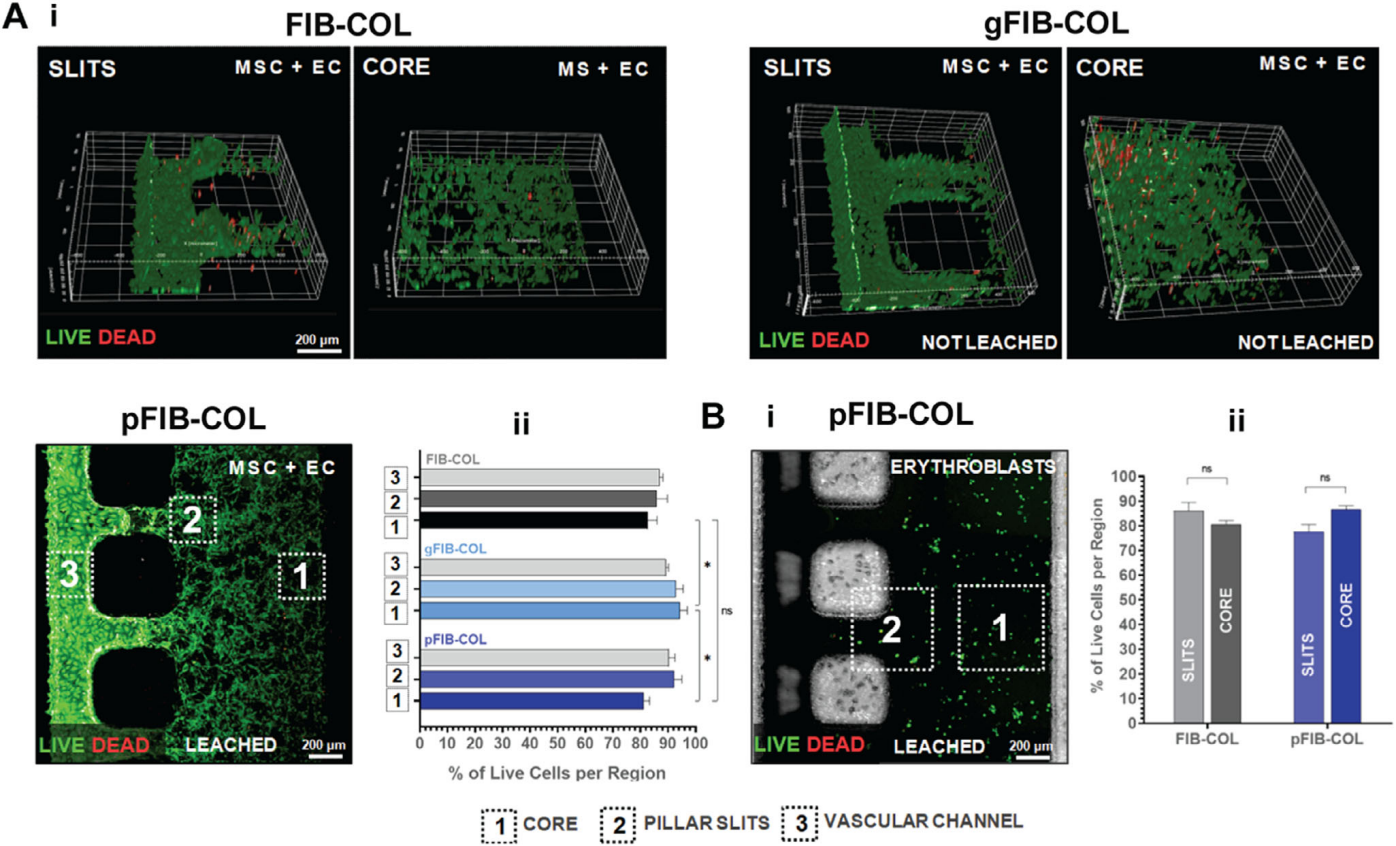
The incorporation and leaching of ALG µGels from the FIB‐COL hydrogel do not compromise cell viability. A) (i) Representative 3D reconstructions or max projections of live‐dead assay images showing the viability of BM‐MSCs and HUVECs upon co‐culture within unmodified FIB‐COL, gFIB‐COL (with 4k ALG µGels), or pFIB‐COL hydrogels for 48 h inside the chip. (ii)Quantification of cell viability (live‐dead assay) in multiple stack images in representative regions of the^[^ ^1^ ^]^ vascular channel,^[^ ^2^ ^]^ hydrogel close to the pillar slits, and^[^ ^3^ ^]^ hydrogels core regions. B) (i) Max projection image of live‐dead assay of differentiated human erythroblasts cultured within pFIB‐COL hydrogels after 48 h inside the chip. (ii)Quantification of erythroblast viability observed in multiple stack images in representative regions of^[^ ^1^ ^]^ the hydrogel core and^[^ ^2^ ^]^ the hydrogel next to the pillar slits. All results are expressed as the mean ± SEM (n = 3 distinct square regions of interest (ROI) per zone (SLITS or CORE) per chip integrating more than 120 counted individual cells (across an image z‐stack of 200 µm). Statistical analysis of % cell viability per each ROI was performed by 2‐way ANOVA with post hoc Turkey's comparison test. * (*p* < 0.05).

Having demonstrated the feasibility of using sacrificial µgels to create porous pFIB‐COL, we next evaluated the cytocompatibility of this approach in 3D cultures of different cell types. We first assessed the viability of human BM‐derived MSCs and human umbilical endothelial cells (HUVECs) (**Figure** **6**A) after 2 days of on‐chip culture, within three hydrogel conditions: FIB‐COL, gFIB‐COL (i.e., with µgel integration), and pFIB‐COL (i.e., after µgel leaching). The vascular channel was also seeded with BM‐MSCs and HUVECs (1:10 ratio) to promote formation of an endothelial monolayer along the channel walls, mimicking a blood vessel interface. No significant differences in cell behavior were observed among the different hydrogels. Cells uniformly colonized the chip, maintaining high viability (> 80%) across multiple regions, including: (1) the distal/core region of the BM compartment, where hydrogel‐embedded cells formed interconnected multicellular networks; (2) the pillar slits, where the two compartments interface; and (3) the vascular compartment, where HUVECs established a cohesive monolayer lining the channel walls (Figure 6A). Similar results were obtained with hydrogel‐embedded erythroblasts, a more sensitive cell type, which showed high viability across multiple spots/z‐stacks per chip (Figure 6B). Collectively, these findings demonstrate that neither the integration of ALG µgels into FIB‐COL to form gFIB‐COL nor their selective enzymatic/chemical leaching to create pFIB‐COL compromises cell viability.

### 2.8 The pFIB‐COL Hydrogels Support the Formation of Primitive Vascular Networks

**Figure 7:**
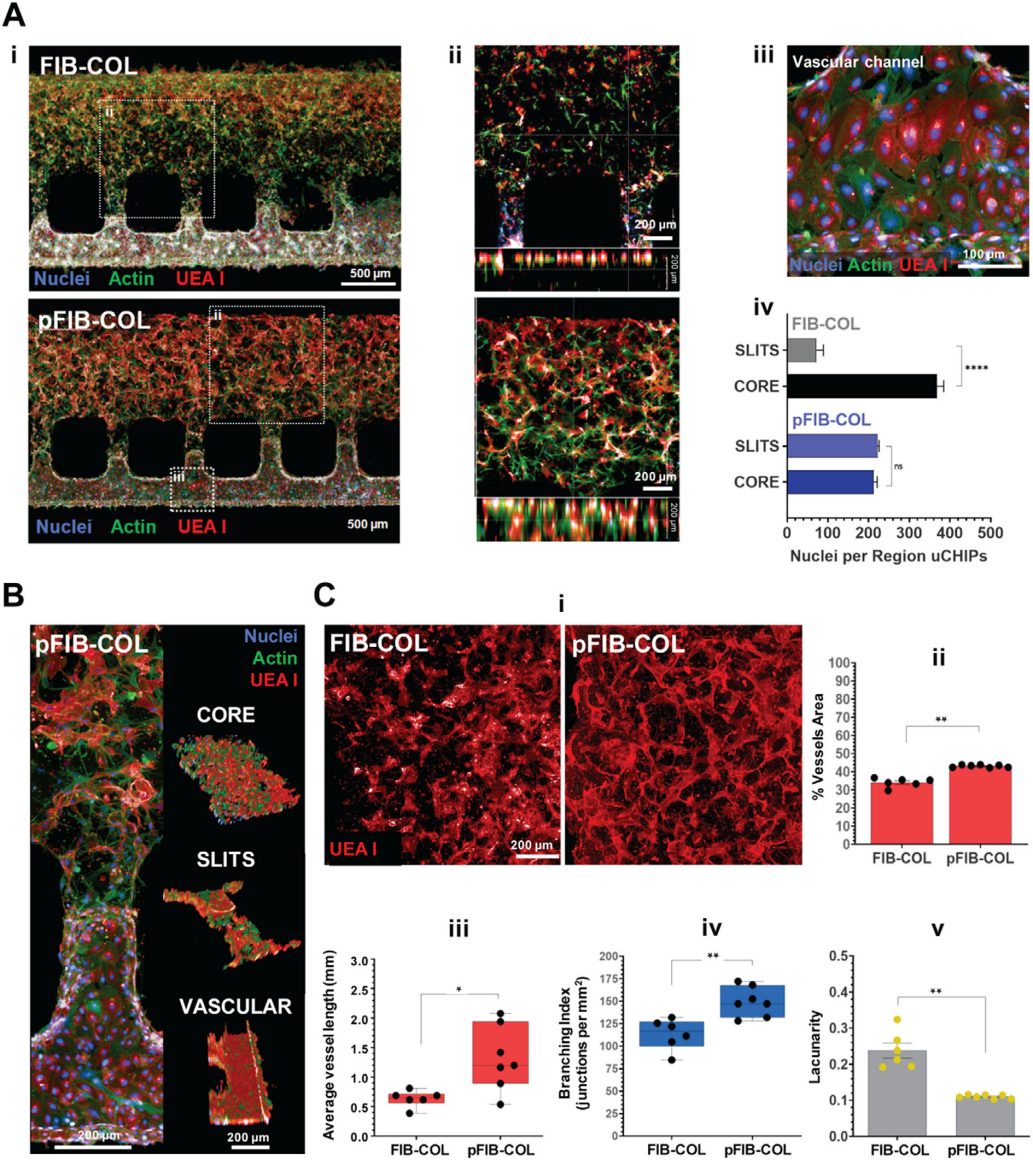
The pFIB‐COL ensures more uniform cell distribution inside the chip and facilitates the establishment of primitive vascular networks. A) HUVECs and BM‐MSCs were co‐cultured within FIB‐COL or pFIB‐COL hydrogels on‐chip under perfusion for 48 h. (i) Representative maximum projection images (actin in green, UEA I in red, nuclei in blue) Scale bar, 500 µm. (ii) Orthogonal cross‐sectional images representing x, y, z cell distribution. (iii) Detail showing the endothelial monolayer in the vascular channel wall. (iv) Image‐based quantifications of the cell nuclei number at slit and hydrogel core in different regions of the chip. B) Insert of image A‐i in pFIB‐COL condition, highlighting the cell organization within the porous hydrogel across one pillar slit of the chip and the corresponding 3D reconstruction at the hydrogels’ core, near the pillar slits, and at the vascular channel. C) i. Confocal imaging showing a detail of microvascular networks within FIB‐COL and pFIB‐COL hydrogels. (ii–v) image‐based quantifications of the vascular morphometric parameters from representative images of the hydrogels core regions (white dotted squares) in the chip highlighted in A. All quantifications are presented for unmodified FIB‐COLand pFIB‐COL hydrogels. All results are expressed as the mean ± SEM (n = 3 distinct square regions of interest (ROI) per zone (SLITS or CORE) per chip integrating more than 120 counted individual nuclei/cells (across an image z‐stack of 200 µm). Statistical analysis of number of counted cell nuclei per chip hydrogel region was performed by 2‐way ANOVA with post hoc Sidak‘s comparison test. For primitive vascular network analysis, 6–7 max‐projection images per chip condition were analyzed in AngioTool for multiple parameters. Statistical analysis comparing vascular network features developed within FIB‐COL versus pFIB‐COL was performed with Mann‐Whitney test. * (*p* < 0.05), ** (*p* < 0.01), and **** (*p* < 0.0001).

We next investigated how co‐culturing BM‐MSCs and HUVECs in pFIB‐COL hydrogels influenced cell distribution, morphology and primitive vascular network formation compared to standard FIB‐COL (**Figure** **7**). Confocal imaging after 48 h of on‐chip co‐culture revealed that, in FIB‐COL hydrogels, cells tended to accumulate in the distal region of the BM compartment (Figure 7A i,ii). A detail of the endothelial monolayer established in the vascular channel is shown in Figure 7A‐iii, highlighting its structural integrity and the characteristic honeycomb organization of the cells. Quantitative analysis (Figure 7A‐iv) revealed that the core hydrogel region contained significantly more cells than areas adjacent to the pillar slits. This accumulation may result from the slight tilt of the chip during hydrogel crosslinking, a necessary step to prevent precursor solution from leaking into the vascular channel. In contrast, no such differences were observed in pFIB‐COL, suggesting that the µgels present in the gFIB‐COL precursor help stabilize cell position, reduce sedimentation during crosslinking, and promote a more uniform distribution throughout the bulk hydrogel. A similar trend was seen along the z‐axis: in FIB‐COL, cells primarily accumulated within the first 50 µm above the glass slide, whereas in pFIB‐COL sedimentation was not detected. (Figure 7A‐ii). Cell sedimentation is a well‐known challenge in hydrogel systems, where cells can settle within the less viscous precursor solution before crosslinking, resulting in uneven distribution. This issue is often overlooked when imaging is limited to shallow depths or when data are presented as maximum‐intensity projections, which merge multiple layers and obscure the true 3D distribution. By imaging the entire chip, we clearly show that pFIB‐COL hydrogels overcome this limitation, promoting uniform cell distribution throughout the matrix and preventing excessive accumulation in any single region (Figure 7B). Importantly, pFIB‐COL supported more robust primitive vascular network formation than FIB‐COL (Figure 7C). Automated AngioTool analysis showed that endothelial cells in pFIB‐COL exhibited greater vascular network coverage, longer average vessel lengths, higher branching indices and lower lacunarity (a measure of network uniformity) compared with FIB‐COL (Figure 7Cii–v), consistent with enhanced endothelial functionality.

In summary, we demonstrate that our strategy enables the generation of a uniform and interconnected porous network within the hydrogel compartment of an organ‐on‐chip device. This architecture lowers flow resistance, improves particle and cell transport between channels, and minimizes cell sedimentation compared with standard FIB‐COL hydrogels. It also supports on‐chip cell organization, including the formation of primitive endothelial networks, underscoring the ability of pFIB‐COL to provide a robust and biomimetic 3D microenvironment. The versatility of this approach allows straightforward adaptation to other hydrogel systems, making it a valuable tool for organ‐on‐chip applications, including advanced infection modeling. Nevertheless, optimization of µgel size and content will be required for different hydrogel compositions and intended applications, and the efficiency of µgel leaching must be assessed across diverse device geometries and under varying flow conditions. Furthermore, future studies should include the evaluation of porous‐architecture stability and hydrogel degradation dynamics over time, as well as in‐depth analysis of long‐term cell behavior and phenotypic stability within the porous matrix. Finally, experiments using clinically relevant pathogenic agents (e.g., viruses and parasites) will be essential to validate the platform beyond model particles and cells.

## 3 Conclusions

Our study establishes sacrificial granular hydrogels as a robust, scalable, and reproducible strategy for engineering porosity, permeability, and fluid dynamics in organ‐on‐chip hydrogel systems. To demonstrate the feasibility of our strategy, we fabricated a simple and economical 3D‐printed model of the BM. By incorporating sacrificial ALG µgels within a FIB‐COL matrix loaded into the hydrogel compartment, we successfully generated pFIB‐COL hydrogels with interconnected void spaces, significantly enhancing permeability and particle transport while preserving the structural integrity of the native hydrogel. This approach also ensured a more uniform distribution of embedded cells and supported the formation of primitive vascular networks, key features for developing ultra‐high‐density culture systems. These advancements highlight the potential of sacrificial granular hydrogels to overcome long‐standing limitations in biomimetic hydrogel models, opening new avenues for refining complex physiological systems. Their integration into organ‐on‐chip platforms could significantly improve the accuracy and accessibility of next‐generation disease models for studying host‐pathogen interactions and therapeutic interventions in infections of the bone marrow.

## 4 Experimental Section

### Production of Alginate µgels

Ultrapure (UP) sodium alginate (PRONOVA UP LVG, Novamatrix, FMC Biopolymers), characterized by an average molecular weight of 190 kDa and a high guluronic acid content (≈70%) was used for most experiments. For the leaching studies, ALG was modified withN‐(1R,8S,9s)‐bicyclo[6.1.0]non‐4‐yn‐9‐ylmethyloxycarbonyl‐1,8‐diamino‐3,6‐dioxaoctane (BCN‐amine, Sigma‐Aldrich) by carbodiimide chemistry and then labeled with a fluorescent azide‐tag via strain‐promoted‐azide alkyne cycloaddition (SPAAC), as previously described.^[^ ^17^, ^29^ ^]^ To produce µgels, sterile ALG solutions (1–3% w/v in 0.9% NaCl)were extruded through a Var J17 encapsulation device (Nisco, Switzerland) equipped with a 0.09 mm nozzle, under a coaxial airflow, into a 0.2 M calcium chloride (CaCl_2_, Sigma‐Aldrich) solution under isotonic conditions. After 10 min, µgels were washed twice in HBSS and finally rinsed in culture medium.

### Preparation of Granular and Porous FIB‐COL Hydrogels

To prepare granular gFIB‐COL hydrogels, a fibrinogen solution (Sigma) was prepared in 0.9% (w/w) NaCl, combined with aprotinin (Sigma) and ALG µgels, and then diluted in endothelial cells growth medium to achieve final concentrations of 5 mg mL^−1^, 25 µg mL^−1^ and 200–4000 µgels µL^−1^, respectively. This mixture was blended with thrombin (0.5 U mL^−1^) and Rat‐Tail Collagen I (0.2 mg mL^−1^) at a 3:1 (v/v) ratio, and allowed to crosslink at 37 °C for 30 min. To produce porous pFIB‐COL hydrogels, the ALG µgels were leached from the gFIB‐COL hydrogels structure through incubation with 100 U mL^−1^ of alginate lyase, with or without 50 mM of sodium citrate, at 37 °C in a CO_2_ incubator under agitation for 30 min and washed 2× with PBS prior adding fresh media.

### Design and Fabrication of the BM‐on‐Chip Device–Stereolithography Fabrication of Device Molds

Mold designs for the devices were created using Fusion 360 (Autodesk) and fabricated with a Form 3B SLA printer (Formlabs) through the PreForm software interface provided by Formlabs. Printing was performed using Grey Pro proprietary resins from Formlabs. The computer‐aided design (CAD) files for all organ chip molds, along with a detailed list of their dimensions, are provided as downloadable resources in the Supporting Information

### Postprocessing of Printed Molds

After printing, the molds were cleaned by submerging them in isopropyl alcohol (IPA) for 20 min using a FormWash system (Formlabs). Once washed, the molds were air‐dried until all traces of IPA had evaporated. Subsequently, the molds were cured under UV light in a FormCure unit (Formlabs) at 60 °C for 15 min for clear resin parts and at 80 °C for 30 min for grey resin parts. After the curing process, the molds were cleaned with water and soap to remove any remaining solvent or chemical that could affect the PDMS polymerization. To address warping induced by the printing and curing stages, the molds were heat‐treated at 100 °C for 6 h. After removal from the oven, the molds were clamped between the blocks and allowed to cool down to room temperature.

### Fabrication of PDMS Layers via Soft Lithography

PDMS (Sylgard 184, Ellsworth Chemical) was prepared by combining the curing agent and elastomer base at a 1:10 weight ratio, followed by degassing to remove air bubbles. PDMS mixed with curing agent was then poured and cast onto the GreyPro resin master molds and allowed to cure at room temperature for 24 h.

### Assembly of the Microfluidic Device

Once cured, the PDMS layers were extracted from the molds. Dust was removed from the PDMS surfaces with clear packing tape. Openings and through‐holes were created as needed using biopsy punches. The devices were finally closed by covalent bonding to a flat PDMS layer or a glass cover slide, using Oxygen plasma treatment.

### Determination of Hydrogels’ Fluidic Resistance and Permeability

The fluidic resistance and permeability were determined for FIB‐COL and pFIB‐COL (gFIB‐COL with 2000 µgels µL^−1^ + leaching) hydrogels. The experimental setup consisted of a reservoir containing cell culture medium positioned at a fixed height *h*, connected via silicone tubing (length *lt_1_* = 40 cm, radius *rt* = 0.25 mm) to a hydrogel‐loaded chip. After passing through the hydrogel, the medium flowed through a second tubing segment (length *lt_2_* = 3 cm) into a serpentine chip, where the flow rate was measured (Figure 4A).

Fluidic resistance was calculated using the fluidic analogue of Ohm's law:

(3) P=R·Q

where the pressure drop (*P*) was determined by the reservoir height, and the flow rate (*Q*) was derived from experimental measurements.

The hydrogel was housed within a PDMS‐PDMS chip with a square cross‐section (width *w* = 1 mm, height *b* = 1 mm, length *L* = 30 mm). The serpentine chip consists in a PDMS‐PDMS construct with a square cross‐section (*w* = 1 mm, *b* = 1 mm, total length *L* = 218.85 mm), featuring five straight segments (each *L_s_* = 40 mm) to enable multiple measurements of the fluid front position over time. Assuming an incompressible fluid and conservation of mass, the flow rate (*Q*) remained constant throughout the system. The absence of hydrogel in the serpentine chip allowed clear visualization of the fluid front post‐hydrogel traversal, critical for precise tracking of fluid advancement.

Fluid front progression was recorded using a high‐resolution Raspberry Pi camera (12.3 MPx, Sony IMX477 sensor) equipped with a 20 mm focal length microscopic lens (Waveshare 100× Industrial Microscope Lens, C/CS Mount) and a CSI‐USB UVC board. Top‐view images of the serpentine chip were captured, and the distance travelled by the fluid front (in pixels) was measured using ImageJ software (Figure 4Aiii,iv). Pixel measurements were converted to micrometers using the channel width (*w* = 1 mm) as a reference.

Fluid front velocity (*v*) was calculated as:

(4) v=Δx/Δt

where *Δx* is the positional change of the front, and *Δt* is the time interval between consecutive images. The flow rate was then determined using:

(5) Q=b·w·v

where *b* and *w* represent the channel height and width, respectively.

Flow was driven by hydrostatic pressure (*P*), generated by elevating the medium‐filled reservoir to a height (*h*) using a retort stand. The pressure was calculated as:

(6) P=ρ·g·h

where *ρ* is the density of the cell medium and *g* is the gravitational acceleration constant (9.81 m ^−1^s^2^). By varying the reservoir height, different pressure values were applied, yielding corresponding flow rates. Across a broad pressure range, a linear relationship between pressure (*P*) and flow rate (*Q*) was observed, satisfying the fluidic Ohm's law (*P = R ⋅ Q*). Linear regression of the plotted data provided the system's fluidic resistance (*R*).

The resistance of the tubing was calculated using the cylindrical tube resistance formula:

(7) $$R_{\mathrm{tube}}=\left(8\eta l_{t}\right)\;/\;\left(\pi \;{r_{t}}^{4}\right)$$

where *η* is the medium viscosity and *l_t_* is the total tubing length. The tubing resistance was found to be negligible compared to the experimentally determined system resistance, confirming that the hydrogel's structure dominated the overall resistance.

Permeability (*K*) was then derived using the equation:

(8) K=(η·L)/(S·R)

where *S* is the hydrogel channel's cross‐sectional area (*S = w ⋅ b*).

Error Analysis

Pressure uncertainty (*δP*) was estimated based on the error in reservoir height (*δh*), using:

(9) δP=ρ·g·δh

Multiple images at a single pressure were analyzed to calculate the standard deviation of the fluid front distance (*Δx*), yielding the velocity error (*δv*). Flow rate error was computed as:

(10) δQ=b·w·δv

The fluidic resistance error (*δR*) was derived from linear regression, and permeability error (*δK*) was calculated via error propagation:

(11) $$\delta K=\left(\eta \cdot L\cdot \delta R\right)\;/\;\left(S\cdot R^{2}\right)$$

### Permeation Studies Using Model Particles

To assess model particles' permeation through the hydrogels, the microfluidic system's baseline fluorescence was first established. Subsequently, fluorescently‐labelled model particles (200 nm (green) or 2 mm (red) carboxylate‐modified fluorescent microspheres, 500 µL, 1:1000 in HBSS, FluoSpheres, ThermoFisher Scientific), or 1 × 10^6^ human RBCs (≈8 mm) pre‐labelled with cell tracker green were introduced into the channels. These were delivered through the device's inlet, and flow was initiated setting flow at 10 µL per min using a syringe pump with 5 min on‐off cycles for 30 min followed by tilting the device setup to leverage gravitational pull toward the basal side of the bone‐marrow compartment over 24 h. For pre‐labelled RBCs, flow was initiated setting flow at 10 µL per minute for 5 min using a syringe pump, stopped, imaged for early time‐point, and re‐initiated with continuous flow during 1 h. Immediately after the perfusion period, the system was imaged using a Leica TCS SP5 AOBS confocal microscope (Leica Microsystems, Germany) or a high‐content imaging system equipped with an automated widefield/confocal fluorescence microscope (Opera Phenix Plus, Revvity).

### Cell Culture

HUVECs (Lonza) were cultured in ECs growth medium as previously described, which consisted of MCDB‐131 basal medium supplemented with 5% (v/v) fetal bovine serum (FBS), 1% (v/v) penicillin‐streptomycin (Pen/Strep), 2 mM L‐glutamine, 1 µg mL^−1^ ascorbic acid, 0.2 µg mL^−1^ hydrocortisone, 2 ng mL^−1^ VEGF, 20 ng mL^−1^ IGF‐1R, 10 ng mL^−1^ FGF, and 5 ng mL^−1^ EGF.^[^ ^30^ ^]^ These cells were utilized between passages 5 and 9. Primary human Bone Marrow Mesenchymal Stromal/Stem Cells (BM‐MSC) (Lonza) were grown in α‐MEM medium supplemented with 10% (v/v) MSC‐qualified FBS and 1% (v/v) Pen/Strep. These cells were used between passages 5 and 10. Both cell types were routinely maintained at 37 °C in a humidified environment with 5% (v/v) CO_2_ and were sub‐cultured when reaching ≈80% confluence. The medium for HUVECs was refreshed every 2 days, while that for BM‐MSCs was replaced every 3 days.

Human erythroblasts were derived from in vitro differentiation of human CD34⁺ hematopoietic stem cells (HSCs) obtained from Lonza. Briefly, CD34⁺ cells were cultured for 8 days in StemSpan SFEM‐II (StemCell) supplemented with 1% (v/v) Pen/Strep, hydrocortisone (10^−6^ M, Sigma‐Aldrich), interleukin‐3 (IL‐3, 5 ng mL^−1^, R&D Systems), stem cell factor (SCF, 100 ng mL^−1^, PeproTech), and erythropoietin (EPO, 4 IU mL^−1^, R&D Systems), allowing for both expansion and differentiation into erythroblasts. Differentiated erythroblasts were cryopreserved in liquid nitrogen for later use.

For chip experiments, thawed erythroblasts were maintained in 2D culture for 2–3 days before chip seeding. Cells were cultured in StemSpan SFEM‐II supplemented with EPO (3 U mL^−1^), SCF (60 ng mL^−1^, PeproTech), aprotinin (12.5 µg mL^−1^), and growth factors including hFGF‐B, VEGF, R3‐IGF‐1, hEGF, ascorbic acid, and heparin (all from SingleQuots) and 10% heat‐inactivated human serum was included to support cell viability. Cultures were maintained at 37 °C with 5% CO_2_.

Human blood samples were obtained in agreement with the principles of the Declaration of Helsinki. Human red blood cells (RBCs) were isolated from surplus buffy coats from healthy blood donors. These were kindly provided by the Immuno‐hemotherapy Department of Centro Hospitalar São João (CHSJ) from Porto, Portugal. This is covered by the ethical approval of the service, under which blood donors give informed written consent for the byproducts of their blood collections to be used for research purposes (Protocol reference 90/19). In brief, peripheral RBCs were harvested from the lower fraction of buffy coats following centrifugation (1200 g, 30 min, room temperature (RT), no brake) after the removal of peripheral blood mononuclear cells (PBMCs) and platelet‐rich plasma. The RBCs were then transferred to a fresh 50 mL tube, resuspended in PBS 1× and washed three times by centrifugation at 500 g at RT before being stained with cell tracker green (5 µM, Thermofisher) as per manufacturer instruction, before infusion in home‐made PDMS‐Glass µchips.

### 3D Cell Culture in FIB‐COL Hydrogels

For cell culture experiments, a total of 600–2400 cells per µL (BM‐MSCs, HUVECs, or erythroblasts) were incorporated into the fibrinogen solution, blended with thrombin (0.5 U mL^−1^) and Rat‐Tail Collagen I (0.2 mg mL^−1^) at a 3:1 (v/v) ratio, (with or without added µgels)and allowed to crosslink at 37 °C for 30 min. Cell viability in FIB‐COL and in gFIB‐COL hydrogels before and after µgels leaching (pFIB‐COL) was evaluated immediately upon 3D culture using the Live‐Dead assay. To prepare the staining solution, 1 µL of Ethidium Homodimer‐I (2 mM, dissolved in a 1:4 DMSO:distilled water mixture) and 1 µL of Calcein (1 mg mL^−1^) were combined with 1 mL of culture medium. The cells were then exposed to this mixture for 30 min under standard conditions (37 °C, 5% CO_2_). Following incubation, the staining solution was removed, and the cells were rinsed with fresh culture medium. Imaging was conducted using a Leica TCS SP5 II laser scanning confocal microscope, with consistent settings applied to detect green fluorescence (Calcein, indicating live cells) and red fluorescence (Ethidium Homodimer‐I, indicating dead cells).

### Fluorescence‐Labelling Studies

Cell‐laden hydrogels were analyzed as whole‐mounted samples at predetermined time points (days 1, 3, 5, and/or 7, depending on the experimental design). Samples were rinsed with HBSS and fixed in 4% (w/v) paraformaldehyde (PFA) for 30 min at RT. Following fixation, the gels were permeabilized with 0.25% (v/v) Triton X‐100 for 30 min and blocked with 2% (v/v) bovine serum albumin (BSA) in HBSS1× for 1 h at RT. Samples were then incubated ON with the following staining reagents diluted in 1% (w/v) BSA: Flash Phalloidin Green 488 (1:1000, Biolegend), Rhodamine‐labelled Ulex Europaeus Agglutinin I (UEA I, 1:400, Vector Laboratories). After incubation, samples were washed with 0.05% (v/v) Tween‐20 in HBSS and incubated (or not) for 4 hat RT with Alexa Fluor 647 NHS Ester (Thermo‐Fisher, 1:50) for total protein amide staining. Fluorescence images were acquired using either a Leica TCS SP5 AOBS spectral confocal microscope (Leica Microsystems, Germany) or a high‐content screening system equipped with a fully automated confocal fluorescence microscope (Opera Phenix Plus, Revvity).

### Cryo‐Scanning Electron Microscopy Analysis

Imaging was performed using a high‐resolution scanning electron microscope equipped with X‐ray microanalysis and cryo‐preparation facilities (JEOL JSM‐6301F/Oxford INCA Energy 350/Gatan Alto 2500). Hydrogel samples were rapidly cooled by plunging into sub‐cooled nitrogen (slush nitrogen) and transferred under vacuum to the cold stage of the preparation chamber. Each specimen was fractured, sublimated (“etched”) for 180 s at −90 °C, and coated with Au/Pd by sputtering for 56 + 56 s. The samples were then transferred to the SEM chamber and analyzed at −150 °C using an accelerating voltage of 15 kV.

### Quantitative and Morphological Image Processing

Images were processed using ImageJ software (version 1.50i), while 3D reconstructions were generated with Harmony high‐content analysis software (version 4.8). Measurements of µgels diameter and the number of cell nuclei per imaged section were performed in ImageJ using straight line measurements and analyze particles, respectively. Primitive vascular networks formed within distinct 3D hydrogels were evaluated for vessel percentage area, total number of junctions, and mean vessel length using the AngioTool v 0.6a.^[^ ^31^ ^]^ Specifically, UEA I‐stained images were processed by applying a max projection in ImageJ, followed by integration in AngioTool software with low threshold, vessel thickness, small particles and fill holes filters set at 15, 13, 120, and 0, respectively. For the flow‐based particle tracer studies, multiple lines were plotted in more than 5 pillar slits and the mean fluorescent intensity along these aligned lines were obtained by using ImageJ “Plot Profile.”

### Statistical Analysis

Data analysis was conducted using GraphPad Prism (version 6.0, GraphPad Software Inc.). The normality of the data was assessed with the D'Agostino and Pearson omnibus test. Depending on whether the data followed a parametric or non‐parametric distribution, comparisons between groups were made using Welch's *t*‐test or the Mann‐Whitney test, respectively. For grouped analysis, non‐parametric Kruskal‐Wallis test or two‐way ANOVA with Turkey multiple comparison test was used. A 99% confidence interval was applied, and significant differences were denoted as follows: * (*p* < 0.05), ** (*p* < 0.01), *** (*p* < 0.001), and **** (*p* < 0.0001).

## Conflict of Interest

The authors declare no conflict of interest.

## Supporting information



Supporting Information

## Data Availability

The data that support the findings of this study are available from the corresponding author upon reasonable request.
