# Supplementary material for: Advancing Organ‐on‐Chip Models With a Sacrificial Granular Hydrogel Strategy for Enhanced Permeability and Biomimicry
Source: Small Methods. 2025 Nov 5;9(12):e00652. doi: 10.1002/smtd.202500652 (PMC12716228; doi:10.1002/smtd.202500652)
Supplement: Supplementary file 1 — Supporting Information [file SMTD-9-e00652-s001.pdf]

Advancing Organ-on-Chip Models with a Sacrificial Granular Hydrogel Strategy for Enhanced Permeability and Biomimicry

Hugo R Caires[1], Óscar Castillo-Fernández[2,3,6], Núria Sima[2,4], Mariana V Magalhães[1,5], Andreu Benavent[3,6], Nil Masó[2,6], Wanlapa Roobsoong[7], Carmen Fernandez-Becerra[2,4], Aurora Hernández-Machado[3,6], Hernando A. del Portillo[2,4,8*], Cristina C. Barrias[1,9,10*]

[1]i3S - Instituto de Investigação e Inovação em Saúde, Universidade do Porto, Porto, Portugal,
[2]Institute for Global Health (ISGlobal), Hospital Clínic - Universitat de Barcelona, Barcelona, Spain
[3]Institute of Nanoscience and Nanotechnology (IN2UB), University of Barcelona, Barcelona, Spain
[4]Institut d'Investigació em Ciències de la Salut Germans Trias i Pujol, Badalona, Spain
[5] FEUP - Faculdade de Engenharia da Universidade do Porto, Porto, Portugal
[6]Department of Condensed Matter Physics, University of Barcelona (UB), Barcelona, Spain
[7]Mahidol Vivax Research Unit, Faculty of Tropical Medicine, Mahidol University, Bangkok, Thailand
[8]Institució Catalana de Recerca i Estudis Avançats (ICREA), Barcelona, Spain
[9]INEB - Instituto de Engenharia Biomédica, Universidade do Porto, Portugal
[10]ICBAS-Instituto de Ciências Biomédicas Abel Salazar, Universidade do Porto, Portugal

*Corresponding authors:

ccbarrias@i3s.up.pt
i3S - Instituto de Investigação e Inovação em Saúde, Universidade do Porto, Porto, Portugal, Rua Alfredo Allen, 208, 4200-135 Porto, Portugal

hernandoa.delportillo@isglobal.org
ISGlobal Institute for Global Health, Barcelona and IGTP Institut d'Investigació Germans Trias i Pujol. Ctra. de Can Ruti. Camí de les Escoles, s/n, 08916 BADALONA (Barcelona), Spain

SUPPORTING INFORMATION

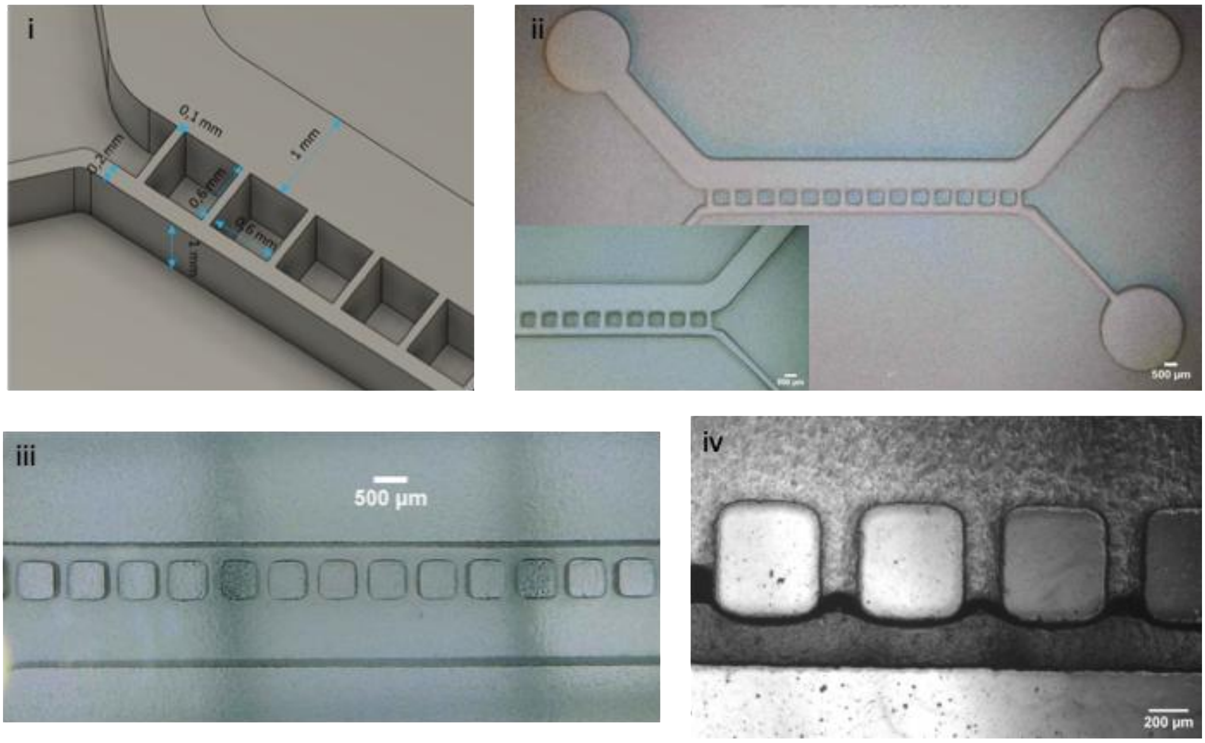

Figure S1. Microfluidic device. i) CAD scheme of a section of the microfluidic device. The design includes a central BM compartment (1 × 1 mm cross-section) and a lateral vascular channel (0.2 × 1 mm cross-section), interconnected by a series of 15 slits (0.1 × 1 mm, 0.6 mm length, spaced 0.6 mm apart). ii) Mold printed with a FormLabs 3+ Printer with GreyPro® resin, iii) Detail of the PDMS cast highlighting the microchannel geometry reproduced from the 3D-printed mold. iv) Detailed view of the FIB-COL hydrogel polymerized inside the BM compartment of the PDMS microfluidic device.
